# Morphological, Ultrastructural, and Histopathological Characterization of Natural *Rhipicephalus annulatus* Infestation in Sheep Skin From Kerbala Province, Iraq

**DOI:** 10.1155/vmi/6736190

**Published:** 2026-09-29

**Authors:** Rana A. Jawad, Zahraa K. Shakir, Fatema Ali Al-Kafhage, Ali Wasfi Sadeq, Azhar Hamzah Hussein, Hiba Alameri, Ihsan Mohammed Sulbi, Afrah Kamil Zabeel

**Affiliations:** ^1^ Department of Internal and Preventive Medicine, College of Veterinary Medicine, University of Karbala, Karbala, Iraq, uokerbala.edu.iq; ^2^ Department of Pathology and Poultry Diseases, College of Veterinary Medicine, University of Karbala, Karbala, Iraq, uokerbala.edu.iq; ^3^ Department of Physiology and Biochemistry, College of Veterinary Medicine, University of Karbala, Karbala, Iraq, uokerbala.edu.iq; ^4^ Department of Surgery and Obstetrics, College of Veterinary Medicine, University of Karbala, Karbala, Iraq, uokerbala.edu.iq; ^5^ Department of Microbiology, College of Veterinary Medicine, University of Karbala, Karbala, Iraq, uokerbala.edu.iq; ^6^ Department of Parasitology, College of Veterinary Medicine, University of Karbala, Karbala, Iraq, uokerbala.edu.iq; ^7^ General Veterinary Hospital, Holy Karbala Governorate, Karbala, Iraq

**Keywords:** hard ticks, Iraq, *Rhipicephalus annulatus*, scanning electron microscopy, sheep, skin histopathology

## Abstract

Ticks constitute most epidemiologically important group of ectoparasites in the veterinary medicine, causing direct host injury and serving as obligate vectors for many pathogenic organisms. The present study was designed for providing an integrated morphological, ultrastructural, and histopathological characterization of *Rhipicephalus annulatus* that are infesting sheep in Kerbala Province, central Iraq. A total of 540 sheep of both sex with variable ages were examined at several rural localities across the province, including Touyrige region, Al‐Husseiniya area, and Al‐Chemmalya countryside, in period between March and November 2023. Tick samples were collected from various anatomical sites on the body of the host. Adult female ticks of genus *Rhipicephalus* were recognized morphologically and exposed to both scanning electron microscopy (SEM) and light histological examination. Gross examination of tick attachment positions revealed local swelling and erythema. Histopathological evaluation of infested skin demonstrated marked epidermal hyperkeratosis, acanthosis, epithelial cell degeneration, multifocal dermal necrosis, and a dense mixed inflammatory infiltrate comprising predominantly eosinophils and lymphocytes. The active immunological response is presented by vascular congestion with dilation at the site of attachment. SEM investigation confirms the specialized mouthparts and cuticular surface adaptations of *R. annulatus* females which enable the mechanical injury and continuous tissue attachment. These findings advance the understanding of host with the ectoparasite connections and underscore the requirement of tick surveillance with control programmed in sheep‐rearing communities in Kerbala Province.

## 1. Introduction

Ticks are blood‐feeding ectoparasites that are abundant among the most important arthropod vectors of vertebrate pathogens globally. They convey a varied spectrum of pathogenic agents, as protozoa, rickettsiae, spirochaetes, and arboviruses, which collectively impose considerable burdens on community and veterinary health [1, 2]. Besides, the climate variations, environmental alteration, and changes in land use have contributed to expansion of tick populations with extended seasonal activity, increasing the environmental distribution of tick and occurrence of tick‐borne sicknesses [3, 4]. In addition to their function as disease carriers, ticks cause direct pathological harm through extended attachment and blood feeding, which results in discomfort, blood loss, decreased productivity, deterioration of the quality of wool and hide, and increase susceptibility to secondary microbial diseases [5, 6].

Amongst ixodid ticks, the species *Rhipicephalus annulatus* (Say, 1821), generally known as the cattle tick, is a single‐host hard tick (Acari: Ixodidae) widely scattered throughout the tropical and subtropical areas [7]. Although cattle are considering its principal host, *R. annulatus* as well invades sheep, goats, and further domestic ruminants, mainly under the mixed grazing systems [8]. Infestation with *R. annulatus* lead to considerable economic losses via blood feeding, skin injury and deterioration, decrease weight gain, and reduced the productivity [9]. Furthermore, *R. annulatus* serves as biological vector of numerous economically vital hemoparasites, including *Babesia bovis, Babesia bigemina*, and *Anaplasma marginale*, making it one of the most significant ectoparasites influencing the production of livestock in endemic regions [10, 11]. Particularly in mixed‐production situations where several livestock species are kept under mixed grazing conditions, *R. annulatus’s* ability to parasitize nonbovine hosts has major implications for disease circulation and transmission. These methods of farming, which are prevalent in the Middle Euphrates region of Africa and Iraq, increase the risk of tick‐borne illnesses by facilitating tick distribution among vulnerable hosts [12, 13].

Complicated structural and immunological reactions are triggered by the tick’s long attachment and blood feeding at host‐tick interface site, a biological environment that is continually changing. Anticoagulants, vasodilators, immunomodulators, complement inhibitors, and anti‐inflammatory proteins are secreted from salivary bioactive compounds that reduce the host defense systems and enable extended attachments; these molecules are important for successful feeding [14, 15]. Therefore, tick infestation causes progressive pathological changes in the skin, including epidermal hyperplasia, degeneration of keratinocyte, vascular congestion, dermal edema, disorganization of collagen, infiltration of inflammatory cells, and tissue remodeling [5, 16]. Although traditional histopathology represents the cornerstone for assessing these abnormal changes, scanning electron microscopy (SEM) offers high‐resolution imagining for the external morphology, mouthparts, attachment structures, and cuticular features that are important for exact species identification and better understanding of parasite–host relations [17, 18]. Consequently, mixing gross morphology, along with ultrastructural examination, and histopathological study offers an inclusive method for associating of parasite morphology with the severity of injury and the improving of our understanding of the pathological mechanisms that are associated with natural tick infestation. In this regard, sheep farming represents an indispensable part of agricultural sector in Kerbala Province, Iraq, greatly contributing to local food security by production of meat, dairy, and wool. Although widespread occurrence of tick infestations in Iraqi livestock has been documented, the widespread studies that combine morphological identification, ultrastructural analysis via SEM, and careful histopathological assessment of skin lesions attributed particularly to *R. annulatus* infestations in sheep are limited. The absence of cohesive pathological characterization data restricts an accurate understanding of host–parasite relationships in natural environment. Therefore, the present study aimed to characterize the gross and ultrastructural morphology of adult female *R. annulatus* collected from naturally infested sheep in different localities of Kerbala Province, Iraq, and investigate the associated histopathological alterations in infested skin obtained from sheep of different ages and both sexes.

## 2. Materials and Methods

### 2.1. Study Area and Animal Subjects

The investigation was carried out in Kerbala Province (32°36′N, 44°01′E), situated within the Middle Euphrates region of central Iraq. Field surveys were conducted at geographically dispersed rural localities representing a cross section of the province’s sheep‐rearing areas, specifically the Touyrige region to the north, Al‐Husseiniya area to the east, and Al‐Chemmalya countryside to the south (Figure 1). Data and specimens were collected over a 9‐month period extending from March to November 2023.

**FIGURE 1:**
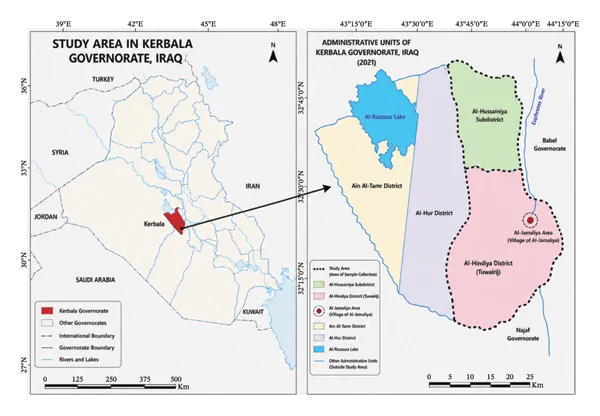
The geographical location of study site in Kerbala governorate, Iraq. The study zone is identified by a black dashed line. The inset map illustrates the geographical location of Kerbala governorate in Iraq, whereas the expanded map shows the sample regions in Al‐Hussainiya subdistrict, Al‐Hindiya district (Tuwairij), and Al‐Jamaliya and Al‐Jamaliya area.

A total of 540 sheep were systematic inspected for an ectoparasitic infestation. Animals of both sexes (males and females) were included in the study population, which consisted of a diverse array of age categories, including lambs and adult animals. In order to facilitate contextual interpretation of infestation parameters, the sex, approximate age group (young, subadult, and adult), effective status (if applicable), and breed for every examined animal were documented.

### 2.2. Tick Collection, Preservation, and Species Identification

Ticks are collected by hand from several anatomical locations on the host’s body, such as the axillary region, inguinal area, perineum, ear folds, and interdigital areas. Only the mature female specimens were selectively conserved for morphological and histological examinations because they represent the principal feeding and reproductive phase and exhibition the most diagnostically significant structural characters. The specimens were washed with physiological saline (0.9% NaCl) to remove all the surface contaminants, at that point stored in 70% ethanol at 4°C until examinations in the laboratory. Morphological identification of samples was conducted by a stereoscopic microscope (Novel, Germany) in agreement with traditional taxonomic keys for ticks that affecting farm animals. Still, the diagnostic standards used for characteristic adult female *R. annulatus* involved the form of basic capitula, palp structure, scutal ornamentation, coxal traits, and arrangement of spiracular plate [8, 19].

### 2.3. Histological Processing of Tick Specimens

External histological characterization was achieved on ten morphologically examined adult female *R. annulatus* samples. Each sample was kept in 10% neutral‐buffered formalin at room temperature for 72–96 h. For uniform sectioning planes, samples were orientated dorsal‐side down in identified tissue cassettes by using spatula after fixation. Standard tissue processing steps included dehydration in graded ethanol (70%, 90%, 95% × 2 for 60 min; 100 × 3 for 45 min), clearing by xylene (three 45 min changes), and paraffin wax infiltration at 60°C (one 30 min immersion followed by three 45 min immersions). The paraffin slices of 5‐µm thick were cut by means of rotary microtome, mounted on clean glass slides, and stained with hematoxylin and eosin (H&E) according to standard procedures [20, 21]. Microscopic inspection with final diagnosis was conducted by a board‐certified veterinary pathologist.

### 2.4. Histopathological Assessment of Infested Sheep Skin

Skin samples were correctly removed from direct tick attachment sites on infested sheep. Age‐ and sex‐matched uninfected sheep from same areas acted as controls, providing reference samples of standard sheep skin structure. Tissue samples were conserved, prepared, cut, and stained by the same H&E practice previously outlined for tick histology. All the histological changes were precisely recorded, and figures were captured at uniform magnifications for comparative valuation.

### 2.5. SEM

Adult female tick samples that were selected for ultrastructural analysis were processed by standard SEM procedures [22]. After fixation in buffered formaldehyde, samples were exposed to dehydration in sequential ethanol series and were dried to maintain their external structure. The desiccated samples were fixed to aluminum stubs using conductive double‐sided carbon adhesive tape and analyzed with a Quanta 450 scanning electron microscope (FEI, USA) at the Electron Microscopy Unit, College of Veterinary Medicine, University of Kerbala. Images displayed the dorsal and ventral surfaces, capitulum, mouthparts, spiracular plates, genital aperture, anal groove, and some diagnostic morphological features were obtained at suitable magnifications for ultrastructural examination.

## 3. Results

### 3.1. Gross Morphology of Adult Female *R. annulatus*

Adult female *R. annulatus* specimens collected from naturally infested cattle exhibited characteristic morphology of hard ticks (Ixodidae). The body shape was elliptical and flattened from the dorsal to ventral surface, including anterior capitulum and wide posterior idiosoma (Figure 2). In engorged females, the idiosoma displayed significant enlargement, but the black scutum was limited to the front dorsal part, a characteristic feature of mature female ixodid ticks. Finally, specimens were recognized as *R. annulatus* species through morphological analysis utilizing traditional taxonomic keys.

**FIGURE 2:**
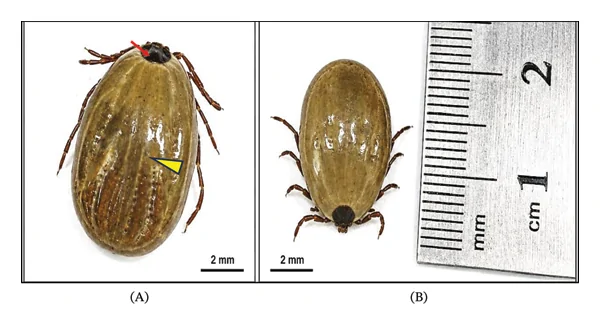
Adult female *Rhipicephalus annulatus* collected from naturally infested cattle. Representative dorsal views of an engorged adult female *R. annulatus* showing the general external morphology. (A) Dorsal view illustrating the oval, dorsoventrally flattened body divided into the anterior capitulum (arrow) and the posterior idiosomic (arrowhead). (B) The same specimen photographed adjacent to a millimeter ruler to indicate the body size. The dark scutum is visible at the anterior dorsal region, while the enlarged idiosoma reflects engorgement following blood feeding. Identification of species was based on the standard morphologic taxonomic keys.

### 3.2. Ultrastructural Morphology by SEM

SEM revealed the external dorsal structure of *R. annulatus* with enhanced detail compared to stereomicroscopy (Figures 3, 4, 5, and 6). The dorsal perspective revealed a clear demarcation between anterior sclerotized scutum and posterior alloscutum, which had significantly enlarged due to blood engorgement (Figure 3). Besides, SEM image provided clear imagining of general body outline and dorsal cuticular surface, facilitating morphologic characterization of this species. Also, higher‐magnification SEM imagination of the anterior region of adult female *R. annulatus* provided full conception of capitulum and associated mouthparts (Figure 4A,B). The dorsal sight established the anterior capitulum in relation to scutum, while the higher‐magnification image discovered the paired palps that surrounding mouthparts. These explanations provided a supporting morphological feature that supported species identification based on external anatomical structures. Investigation of ambulatory legs of adult female *R. annulatus* via SEM revealed robust segmentation with distinct articulations between successive leg parts (Figure 5A,B). The coxa, femur, patella, tibia, and tarsus were distinguished, revealing of characteristic organization of appendages in ixodid ticks. The cuticular surface at higher magnification appeared smooth with scattered surface ornamentation and fine spines visible on segments of legs. These morphological features contribute to structural integrity and mobility of the appendages and also support the identification of *R. annulatus*. Also, SEM examination of the ventral surface of adult female *R. annulatus* showed the genital orifice and the anal groove, which were located posterior to the anus (Figure 6). These ventral structures represent an important external morphological feature that is usually used in taxonomic identification of ixodid ticks.

**FIGURE 3:**
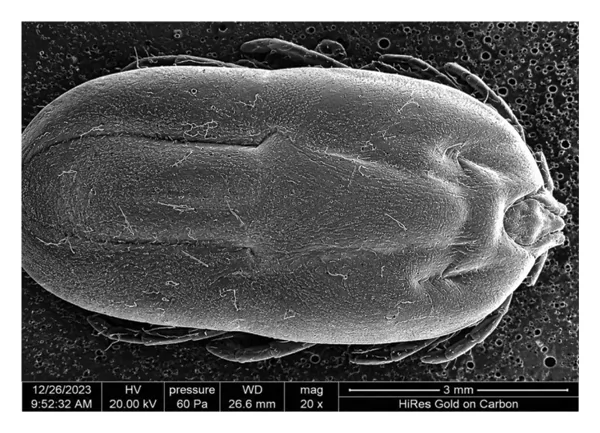
Scanning electron micrograph (SEM) of the dorsal surface of adult female *Rhipicephalus annulatus* displays characteristic oval body shape of engorged female tick, with anterior sclerotized scutum obviously defined from the enlarged posterior alloscutum. Image developed at 20x magnification (accelerating voltage 20.0 kV; working distance 26.6 mm). Scale bar = 3 mm.

**FIGURE 4:**
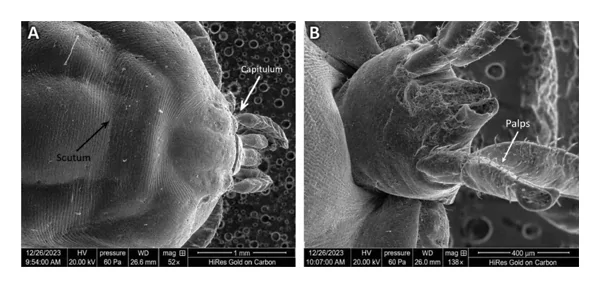
Scanning electron micrographs of capitulum of adult female *Rhipicephalus annulatus*. (A) Dorsal sight of anterior body segment showing the capitulum (white arrow) situated anterior to scutum (black arrow). (B) Enlarged sight of capitulum demonstrating paired palps (white arrow) and external organization of feeding apparatus. These constructions represent the key external morphological characters that used for taxonomic identification of *R. annulatus* species. Scale bars = 1 mm (A) and 400 μm (B).

**FIGURE 5:**
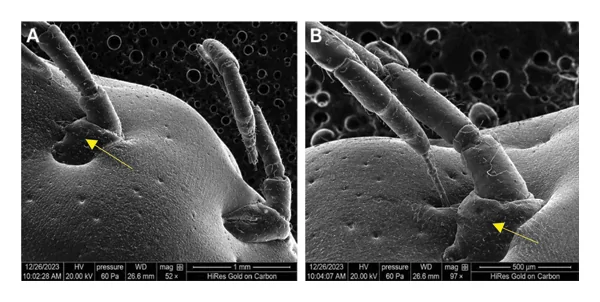
Scanning electron micrographs of ambulatory legs of adult female *Rhipicephalus annulatus.* (A) Dorsolateral view displays the coaxal area (yellow arrow) and proximal segments of ambulatory legs. (B) Higher‐magnification sight showing the segmentation and articulations of the leg, including the coxa, femur, patella, tibia, and tarsus. The cuticular surfaces and external morphology of appendages are clearly noticeable. Scale bars = 1 mm (A) and 500 μm (B).

**FIGURE 6:**
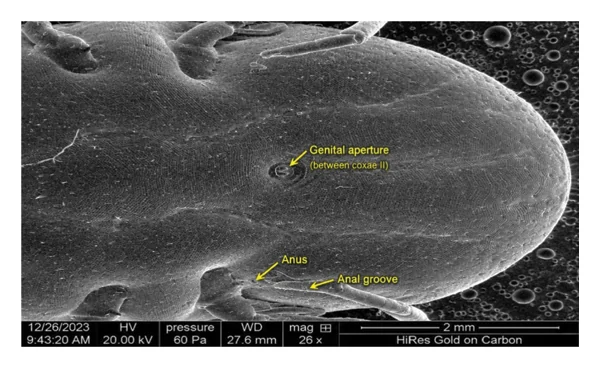
Scanning electron micrograph of the ventral surface of adult female *Rhipicephalus annulatus*. The image shows the genital aperture and the anal groove located posterior to the anus. These ventral external structures are important morphological features used in species identification. Scale bar = 2 mm.

### 3.3. Internal Histological Structure of *R. annulatus*

Histological examination of adult female *R. annulatus* section stained with H&E revealed well‐preserved interior anatomical structures (Figures 7 and 8). At low magnification (Figure 7A), the outer cuticle part appeared as a thick, eosinophilic, complicated exoskeleton surrounding the internal tissues. Underneath the cuticle, skeletal muscle bundles were observed, together with centrally situated synganglion and several midgut diverticula which occupy a large part of the body cavity. At higher magnification (Figure 7B), the cuticle and the underlying skeletal musculature were more clearly resolved, demonstrating the close association between the external body wall and the muscular system that supports locomotion and body integrity (Figure 8A).and B exhibited the anatomical features of the integument and reproductive system. The posterior body wall exhibited the characteristic cuticle with the posterior festoons visible at the margin of the idiosoma (Figure 8A). In adjacent sections, paired tubular structures lined by cuboidal to columnar epithelium corresponding to the oviducts were identified within the posterior body cavity (Figure 8B). These observations further complemented the histological characterization of the adult female and supported the identification of major internal anatomical structures.

**FIGURE 7:**
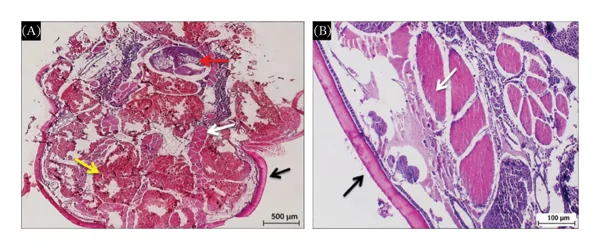
Histological sections of adult female *Rhipicephalus annulatus* stained with hematoxylin and eosin (H&E). (A) Low‐magnification sagittal section showing the multilayered cuticle (black arrow), underlying skeletal muscle (white arrow), synganglion (red arrow), and midgut diverticula (yellow arrow) (H&E, × 4). (B) Higher‐magnification view of the body wall illustrating the multilayered cuticle (black arrow) and underlying skeletal muscle bundles (white arrow) (H&E, × 10).

**FIGURE 8:**
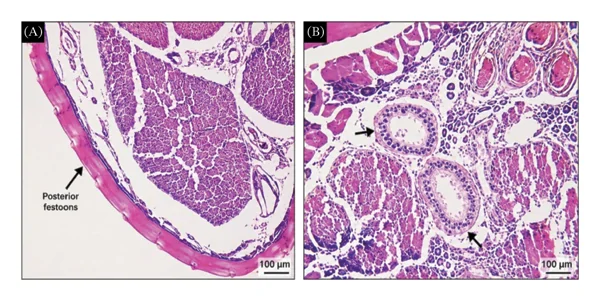
Histological sections of an adult female *Rhipicephalus annulatus* stained with hematoxylin and eosin (H&E). (A) Section through the posterior body region showing the cuticle and the posterior festoons (black arrow) at the margin of the idiosoma (H&E, × 10). (B) Higher‐magnification section showing paired oviducts (black arrows) within the posterior body cavity (H&E, × 10).

### 3.4. Histopathological Alterations in Infested Sheep Skin

Histological examination of H&E‐stained skin sections from uninfected control sheep demonstrated the normal architecture of the skin (Figure 9). The epidermis consisted of a well‐organized keratinized stratified squamous epithelium with a distinct stratum corneum overlying the viable epidermal layers. Beneath the epidermis, the dermis was composed of dense collagenous connective tissue containing numerous hair follicles, associated adnexal structures, and small blood vessels. The deeper aspect of the section showed the transition toward the hypodermis (subcutaneous tissue), preserving the normal structural organization of healthy ovine skin. However, histological examination of skin sections from *R. annulatus* attachment sites revealed marked pathological alterations compared with uninfested control skin (Figure 10). At low magnification, the epidermis exhibited pronounced hyperkeratosis characterized by thickening of the stratum corneum and irregular epidermal hyperplasia (acanthosis). The underlying dermis showed multifocal inflammatory cell infiltration consistent with dermatitis, extending around adnexal structures, including hair follicles. Follicular acanthosis was also evident in affected hair follicles, indicating a localized epidermal proliferative response associated with tick attachment.

**FIGURE 9:**
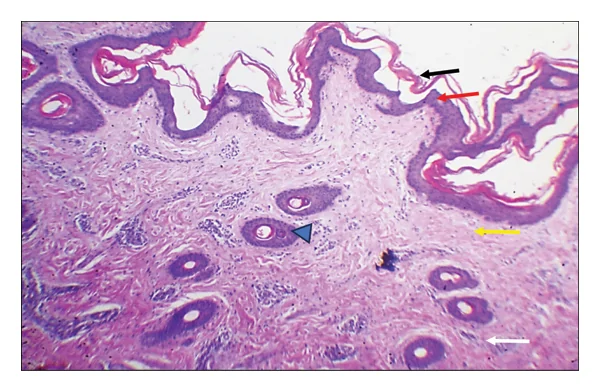
Representative photomicrograph of normal ovine skin demonstrates the normal histological organization of the skin, including the epidermis with a well‐developed stratum corneum (black arrow) and stratum granulosum (red arrow), the underlying dermis (yellow arrow), the superficial hypodermis (subcutaneous tissue) (white arrow), and hair follicles (arrowhead). H&E stain; original magnification × 4.

**FIGURE 10:**
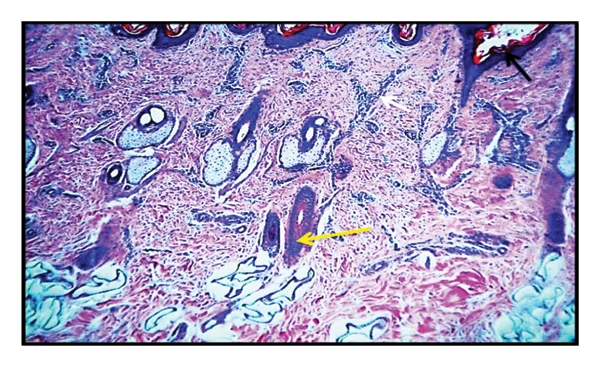
Representative photomicrograph of skin from a sheep naturally infested with *Rhipicephalus annulatus*. The section demonstrates marked epidermal hyperkeratosis characterized by thickening of the stratum corneum (black arrow), multifocal dermal inflammatory cell infiltration consistent with dermatitis (white arrow), and follicular acanthosis characterized by thickening of the follicular epithelium (yellow arrow). Original magnification × 4.

Higher‐magnification examination of the tick attachment site demonstrated marked epidermal hyperkeratosis overlying the affected dermis (Figure 11). Also, the superficial dermis contained focal areas of collagen degeneration characterized by eosinophilic, homogenized collagen bundles with reduced cellularity, accompanied by multifocal inflammatory cell infiltration extending throughout dermal connective tissue. These results are reliable with localized injury and chronic inflammatory variations associated with *R. annulatus* attachment. In addition, higher‐magnification investigation of affected dermis proved a dense inflammatory cell infiltrate dispersed throughout both superficial and mid dermis, with clear perivascular and perifollicular inflammatory cells accumulation (Figure 12). The active inflammatory response linked with *R. annulatus* attachment verified by inflammatory infiltration extended between collagen bundles and the surrounding adnexal structures. However, the hair follicles remained intact but stayed associated with inflammatory infiltrates, while the surrounding dermal connective tissue displayed mild disturbance of its normal structure. These histopathological results are consistent with the chronic dermatitis caused by *R. annulatus* infestation. The arrangements of these lesions were illustrious in animals of both sex and in all age groups studied, with more important changes noted in locations where *R. annulatus* had been attached for prolonged time.

**FIGURE 11:**
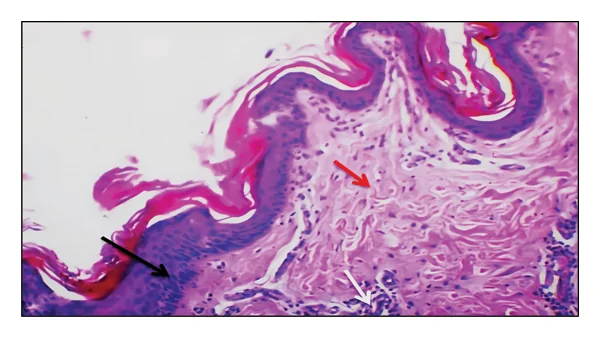
Representative photomicrograph of sheep skin sample that naturally infested with *Rhipicephalus annulatus*. The section shows marked epidermal hyperkeratosis (black arrow), focal degeneration (hyalinization) of dermal collagen bundles (red arrow), and multifocal inflammatory cell infiltration within the dermis (white arrow). Original magnification × 10.

**FIGURE 12:**
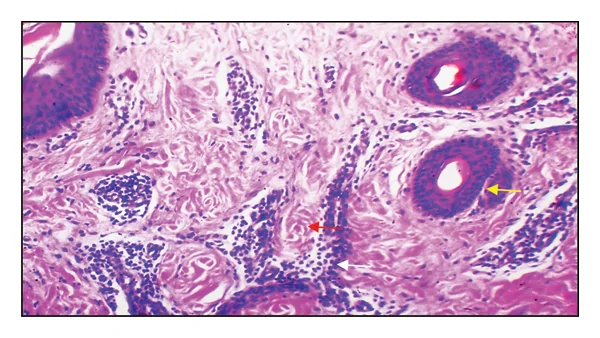
Representative photomicrograph of H&E‐stained skin from a sheep naturally infested with *Rhipicephalus annulatus*. The section demonstrates dense inflammatory cell infiltration within the superficial and mid dermis, with prominent perivascular and perifollicular inflammatory aggregates surrounding hair follicles and extending between collagen bundles, consistent with chronic dermatitis associated with tick infestation. Original magnification × 10.

## 4. Discussion

The existing study offers a comprehensive morphological, ultrastructural, and histopathological description for adult female *R. annulatus* with accompanying dermal lesions in naturally infested animals from Kerbala Province, Iraq. By participating in gross morphological check, SEM, tick histology, and skin histopathology, this study offers an inclusive assessment of both parasite structural features and the host responses under natural field conditions. To our knowledge, such integrated study has not been previously described for *R. annulatus* infesting sheep in this province (Kerbala/Iraq), therefore contributing appreciated baseline data for understanding the host–parasite interactions and tick‐related pathology in Iraqi livestock.

In addition, the morphological examination documented the character of the collected specimens as adult female *R. annulatus*, steady with previous structural and epidemiological studies that identifying this species among chief ixodid ticks invading cattle and small ruminants in Iraq [23, 24]. Above and beyond that, the gross examination and SEM recognized the characteristic morphological features of *R. annulatus*, including inornate scutum, distinct capitulum (Figure 4), segmented legs bearing terminal claws (Figure 5), and ventral constructions as genital aperture, anus, and anal groove (Figure 6). These structures are well‐recognized differences that enable secure attachment of ticks to host with efficient blood feeding, and successful parasitism, reflecting specific hematophagous life of ixodid ticks. Additionally, SEM enabled full visualization of the external morphological and cuticular features, including capitulum, scutum, genital aperture, anal groove, and leg structures, which are important diagnostic structures for identification of species. The morphologic features noticed in the current study are consistent with previous descriptions of *R. annulatus* and demonstration the value of SEM as a reliable tool for full morphological description and correct taxonomic identification of ticks [22, 25, 26].

In addition to the external morphological observations, the internal histological sections of female *R. annulatus* exhibited efficient anatomical structures, including the multilayered cuticle, muscular structure, synganglion, midgut diverticula, and reproductive organs, including oviducts (Figures 7 and 8). The detected organization of the nervous, digestive, and reproductive systems is reliable with previous anatomical studies of cattle ticks and related ixodid species [27]. The identification of oviducts in engorged females is in agreement with the reproductive anatomy of ixodid ticks and reflects the physiological changes associated with blood feeding and reproductive development. Furthermore, the clear preservation and differentiation of internal tissues demonstrate the effectiveness of the histological preparation and staining procedures used in the present study. These findings support the utility of whole‐tick paraffin embedding and sectioning as a reliable approach for detailed anatomical characterization and comparative morphological investigations of ticks [28].

Obviously, compared with the normal histological structure that observed in uninfested ovine skin (Figure 9), skin sections obtained from sheep naturally infested with *R. annulatus* displayed noticeable epidermal and dermal changes, including hyperkeratosis, follicular acanthosis, inflammatory cell infiltration, and focal degeneration of the dermal collagen bundles (Figures 10, 11, and 12). The distinct thickening of the stratum corneum and follicular epithelium noticed in Figure 10 is indicative of chronic epidermal activation and tissue remodeling correlated with prolonged *R. annulatus* attachment and nutritious activity. Similar epidermal and dermal lesions have been described in both cattle and small ruminants which infested with ixodid ticks and are considered distinctive features for the chronic tick‐associated dermatitis [5, 29].

Another notable histopathological finding was the presence of inflammatory infiltrates that was observed throughout the dermis, mainly the prominent perivascular and perifollicular inflammatory aggregation surrounding the hair follicles in Figure 12, suggesting an active host immune response against tick feeding and salivary antigens. The existence of both eosinophils and lymphocytes within the inflammatory infiltrate is indicative of immediate and delayed hypersensitivity responses that are associated with frequent exposure to tick‐derived antigens. As known, eosinophil recruitment is commonly associated with antiparasitic and allergic reactions, whereas the lymphocytic infiltration reflects initiation of antigen‐specific cellular responses following the repeated infestations [30, 31].

In addition to inflammation, the focal degeneration of dermal collagen bundles was observed in affected skin samples (Figure 11). These lesions may result from combined effects of mechanical injury caused by hypostomal penetration and biological activity of saliva compounds released through feeding of *R. annulatus*. This saliva contains a various array of immunomodulatory, anticoagulant, anti‐inflammatory, and proteolytic molecules, which enable prolonged blood feeding while changing the local host immune responses [14, 31, 32]. Moreover, the prolonged exposure to these bioactive salivary particles may contribute to local tissue degeneration, persistence inflammatory lesions, and delayed tissue repairing at sites of attachment. However, the widespread occurrence of *R. annulatus* between sheep sampled from different parts of Kerbala Province, in cooperation with its recover from multiple anatomical positions, indicates the infestation is mostly spread and not limited to specific geographic region or host group. These detected cutaneous lesions might harmfully disturb animal welfare and production capacity by disturbance of skin integrity, increased susceptibility to secondary infections, and chronic inflammations, probably contributive to decreased wool quality, reduced weight gain, and economic losses in sheep [33]. Hence, combined tick‐control methods including repetitive surveillance, strategic acaricide application, and environmental handling practices are extremely recommended to reduce tick infestation pressure and diminishing its impact on animal health and production. Despite provided that novel baseline data on morphological, ultrastructural, and histopathological features of *R. annulatus* infestation of sheep in Kerbala Province, this study has some limits. The study was restricted only to naturally infested sheep from restricted geographical area and focused entirely on adult female ticks. Moreover, molecular confirmation of tick identification and detection of tick‐borne pathogens were outside the scope of the current study. Thus, the results would be interpreted within the situation of the study zone and sampling period. Future studies should involve many geographical regions, for both male and female ticks, and seasonal investigations, and molecular studies are recommended to provide more inclusive understanding of *R. annulatus* biology, spreading, and its role in transmission of tick‐borne pathogens.

## 5. Conclusion

This study reveals that natural infestation of sheep with *R. annulatus* in Kerbala Province, Iraq, is related with discrete morphological, ultrastructural, and histopathological variations. Adult female ticks of *R. annulatus* exhibited characteristic structural adaptations for blood feeding on host and attachment, including well‐developed capitulum, denticulate mouthparts, and claw‐bearing legs, while the histopathological examination of infected skin showed epidermal hyperkeratosis, multifocal dermal necrosis, acanthosis, vascular alterations, and mixed eosinophilic and lymphocytic cell infiltration. These lesions are steady with compound inflammatory and immunological activation triggered by extended tick attachment and nourishing. Collectively, these results offer a wide‐ranging characterization of ectoparasite and related host tissue pathology below natural conditions of field, contributing respected reference line data on *R. annulatus* infestation of sheep in Iraq. The study also highlights the importance of the continuous tick investigation and integrated ectoparasite control program for improving sheep health and productivity and for reduce the problem of tick‐borne diseases in endemic areas.

## Author Contributions

All authors contributed to the conception and design of the study and the preparation of the research methodology. Rana A. Jawad, Fatema Ali Al‐Kafhage, Ihsan Mohammed Sulbi, and Afrah Kamil Zabeel were responsible for field sampling across the localities of Kerbala Province throughout the study period. Zahraa K. Shakir, Hiba Alameri, Ali Wasfi Sadeq, and Azhar Hamzah Hussein performed laboratory data generation, interpreted the results, and drafted the Discussion section. The corresponding author had full access to all study data and assumes responsibility for the integrity and accuracy of the data analysis.

## Funding

This research received no specific grant from any funding agency in the public, commercial, or not‐for‐profit sectors. The study was conducted using institutional resources of the College of Veterinary Medicine, University of Kerbala.

## Disclosure

All authors reviewed and approved the final manuscript.

## Ethics Statement

Zahraa K. Shakir, as the corresponding author and guarantor of this manuscript, affirms that this paper is an honest, accurate, and transparent account of the study reported; that no important aspects of the study have been omitted; and that any discrepancies from the study as planned have been explained.

## Conflicts of Interest

The authors declare no conflicts of interest.

## Data Availability

The datasets generated and/or analyzed during the current study are available from the corresponding author upon reasonable request.
